# Heterogeneity of Cancer-Associated Fibroblasts and the Tumor Immune Microenvironment in Pancreatic Cancer

**DOI:** 10.3390/cancers14163994

**Published:** 2022-08-18

**Authors:** Tomohiko Shinkawa, Kenoki Ohuchida, Masafumi Nakamura

**Affiliations:** Department of Surgery and Oncology, Graduate School of Medical Sciences, Kyushu University, Fukuoka 819-0395, Japan

**Keywords:** cancer-associated fibroblasts (CAFs), pancreatic ductal adenocarcinoma (PDAC), stroma-targeting therapy, tumor microenvironment, cancer immunotherapy, single-cell RNA sequencing, differentiation

## Abstract

**Simple Summary:**

Stroma-targeting therapy in pancreatic ductal adenocarcinoma (PDAC) has been extensively investigated, but no candidates have shown efficacy at the clinical trial stage. Studies of cancer-associated fibroblast (CAF) depletion in a mouse model suggested that CAFs have not only tumor-promoting function but also tumor-suppressive activity. Recently, single-cell RNA sequencing (scRNA-seq) has revealed the complex tumor microenvironment within PDAC, and subpopulations of functionally distinct CAFs and their association with tumor immunity have been reported. However, the existence of tumor suppressive CAFs and CAFs involved in the maintenance of PDAC differentiation has also been reported. In the future, therapeutic strategies should be developed considering these CAF subpopulations, with the hope of improving the prognosis of PDAC.

**Abstract:**

Pancreatic ductal adenocarcinoma (PDAC) is one of the most lethal cancers, with a 5-year survival rate of 9%. Cancer-associated fibroblasts (CAFs) have historically been considered tumor-promoting. However, multiple studies reporting that suppression of CAFs in PDAC mouse models resulted in more aggressive tumors and worse prognosis have suggested the existence of a tumor-suppressive population within CAFs, leading to further research on heterogeneity within CAFs. In recent years, the benefits of cancer immunotherapy have been reported in various carcinomas. Unfortunately, the efficacy of immunotherapies in PDAC has been limited, and the CAF-driven cancer immunosuppressive microenvironment has been suggested as the cause. Thus, clarification of heterogeneity within the tumor microenvironment, including CAFs and tumor immunity, is urgently needed to establish effective therapeutic strategies for PDAC. In this review, we report the latest findings on the heterogeneity of CAFs and the functions of each major CAF subtype, which have been revealed by single-cell RNA sequencing in recent years. We also describe reports of tumor-suppressive CAF subtypes and the existence of CAFs that maintain a differentiated PDAC phenotype and review the potential for targeted therapy.

## 1. Introduction

Pancreatic ductal adenocarcinoma (PDAC) is one of the most lethal cancers, with a 5-year survival rate of 9% [1]. Due to its highly malignant biological profile, PDAC has been the focus of intensive research, but the development of effective therapies for PDAC has been limited compared with other gastrointestinal cancers, for which a variety of molecularly targeted drugs are being developed. The current standard chemotherapy for PDAC is combination therapies consisting of cytotoxic anticancer drugs such as FOLFIRINOX [2] and gemcitabine plus nab-paclitaxel [3]. FOLFIRINOX in combination with leucovorin, 5-fluorouracil, irinotecan, and oxaliplatin has a partial response rate of nearly 80%. However, the selection of these therapies is often based on patient performance status and comorbidities [4], and chemotherapy regimens such as FOLFIRINOX are more toxic than other regimens and cannot be used in older patients with a low performance status. On the other hand, targeted therapy for other solid tumors, such as lung and breast cancer, has been dramatically improved by biomarker selection, which contrasts markedly with PDAC treatment strategies [5,6,7]. Thus, the development of novel therapeutic strategies for PDAC patients is crucial to improve the prognosis of PDAC. Therefore, the development of novel modalities and the identification of targeted therapies for individual patients with PDAC are critical.

PDAC is often resistant to these established chemotherapies, in part because of the high-density stromal tumor microenvironment [8]. Cancer-associated fibroblasts (CAFs) are the major stromal cells within the PDAC and are a source of extracellular matrix (ECM) proteins that create physical and metabolic barriers reducing therapeutic efficacy [9]. Furthermore, CAFs not only promote chemotherapy resistance [10,11,12] but also induce immunosuppression [13] and tumor metabolism [14] and secrete inflammatory ligands [15], which have historically been considered as tumor-promoting components. Accordingly, depletion of CAFs could be a promising therapeutic strategy for PDAC treatment [16,17]. However, stroma-targeting therapies have failed in clinical trials [18]. Nonetheless, selective genetic depletion of a α-smooth muscle actin (αSMA)-positive CAF populations and pharmacological blockade of sonic hedgehog (SHH) signaling were shown to increase the proportion of poorly differentiated PDAC cells and rather increase malignancy [19,20]. These findings led us to speculate that there may be functionally heterogeneous CAF populations in the PDAC stroma. Not only CAFs but also diverse immune cells are present in the PDAC stroma, resulting in advanced heterogeneity.

Recently, the efficacy of cancer immunotherapy has been reported in various carcinoma types, bringing about a major revolution in cancer drug therapy and cancer research. In this global trend, immunotherapy was also expected to be effective in pancreatic cancer, but the efficacy of immune checkpoint inhibitors (for example, anti-CTLA-4 antibodies and anti-PD1/PD-L1 antibodies) currently in clinical use is limited. One of the causes is a complex immunosuppressive microenvironment composed of a variety of immune suppressive cells, such as regulatory T cells (Tregs), tumor-associated macrophages (TAMs), myeloid-derived suppressor cells (MDSCs), and γδ T cells, and CAFs are believed to be involved in the recruitment of these immunosuppressive cells. However, the relationship between CAF heterogeneity and the recruitment of tumor immunosuppressive cells remains unclear.

Single-cell RNA sequencing (scRNA-seq), in which tissue is isolated down to the single-cell level for expression analysis, has become popular and is a powerful tool for functional analysis of the pancreatic cancer microenvironment, with a much higher degree of heterogeneity compared with conventional bulk gene expression analysis [21]. In addition, the results of this comprehensive gene profiling have made it possible to classify conventional cell types into various functional cell populations and to predict the characteristics of each cell population.

In this review, we report a synopsis of the latest findings on the heterogeneity of CAFs and the functions of each major CAF subtype, which have been revealed by single-cell analysis in recent years. We also describe reports of tumor-suppressive CAF subtypes and the existence of CAFs that maintain a differentiated PDAC phenotype, as well as reviewing the potential for targeted therapy.

## 2. Heterogeneity of CAFs in PDAC

CAFs are one of the most abundant cell types within the PDAC stroma, comprising 15–85% of stromal cells [22]. CAFs form a physical and metabolic barrier by providing ECM proteins, reducing the therapeutic efficacy to PDAC [23,24], and have also been thought to promote tumor growth and invasion [17,25,26,27,28]. As in many of these studies, αSMA has historically been considered a hallmark marker in CAFs compared with normal fibroblasts [29]. CAFs used in vivo/in vitro have been identified as α-SMA-positive or Glial fibrillary acidic protein-positive fibroblasts isolated from PDAC tissue by the outgrowth method or by fluorescence-activated cell sorting. This is based on the view of CAFs as a homogeneous group of mesenchymal cells in the tumor microenvironment (TME), all of which are considered to exhibit tumor-promoting functions. However, several studies suppressing CAFs in PDAC mouse models have shown that CAFs may also function to restrain tumors [19,20], suggesting the presence of heterogeneity within CAFs. Since then, several studies have revealed specific markers for various CAF subtypes, including CD10-positive CAFs reported by Ikenaga et al. [30], to label subpopulations of CAF that contribute to cancer progression and resistance to therapy [31]. Although these studies have provided new insights into heterogeneity within CAFs, none of these markers are specific to each subpopulation. A number of CAF markers that have been used to identify CAF subsets overlap in expression, including fibroblast activation protein α (FAPα), podoplanin, CXCL12, fibroblast-specific protein-1 (FSP1/S100A4), platelet-derived growth factor receptor α/β (PDGFRα/β), and periostin [13,32,33]. These markers are also expressed in other normal cell populations, such as pericytes, lymphatic endothelial cells, and fibroblast reticulocytes. Such evidence for the presence of CAF heterogeneity in PDAC has also been provided by immunostaining, in which CAF markers such as αSMA, podoplanin, PDGFRα/β, FSP1, and FAP have also been shown to differ in their staining intensity, distribution, and overlap across tumor tissue [34,35,36]. Öhlund et al. focused on the fact that CAFs located in the proximity of tumor cells express high levels of α-SMA, while CAFs distal to the tumor cells express low levels of α-SMA and instead express high levels of IL6 and demonstrated that α-SMA-high (myofibroblastic CAFs; myCAFs) and αSMA-low/IL6-high (inflammatory CAFs; iCAFs) subpopulations were each located in spatially distinct regions in the same PDAC tissue [37]. Although the heterogeneity of CAFs has thus been clarified, it has been difficult to comprehensively analyze the heterogeneity of CAFs in PDAC tissue and classify them into subpopulations.

**Figure 1:**
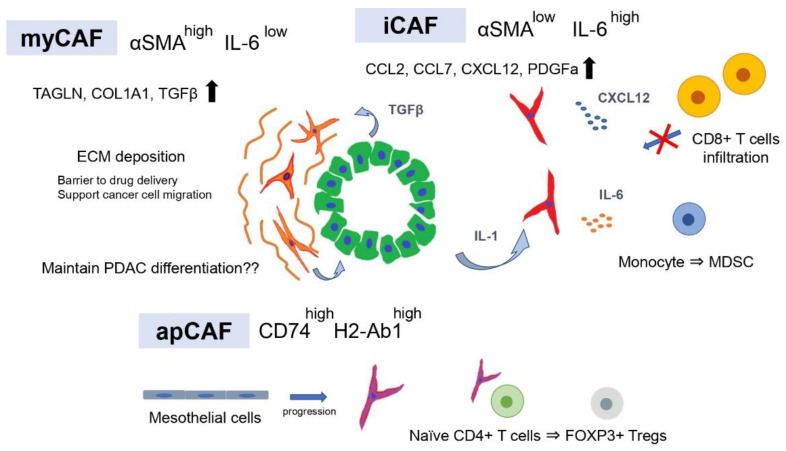
CAF subtypes contribute to the formation of tumor immunosuppressive microenvironments.

Recently, scRNA-seq has been developed and extensively used to examine heterogeneity within human and mouse PDAC tumors. Conventional gene expression profiling using bulk tissue provides limited information to elucidate the heterogeneity in tumor tissues because the data consist of merged expression signatures of individual cells in the tissue. However, scRNA-seq enables gene expression profiling at the single-cell level and has become a powerful tool for understanding cellular heterogeneity within the same PDAC tissue. By scRNA-seq of human and PDAC tissues, Tuveson and colleagues revealed that there are two distinct CAF subtypes in the same PDAC tissue, myCAFs and iCAFs, as shown previously [38] (Figure 1). myCAFs are characterized by high expression of α-SMA and represent many of the classic CAF features, such as secretion of ECM components. In contrast, iCAFs are characterized by low expression of α-SMA and high expression of IL6 in addition to inflammatory chemokines and cytokines involved in immunoregulation. These myCAF and iCAF phenotypes are regulated by two critical factors, TGFβ and IL-1, respectively, and it has been demonstrated that PDAC cells are the source of these factors [38,39,40]. Initial studies on CAFs in PDAC revealed that TGF-β signaling is a fibrosis-promoting pathway [41,42], and myCAFs contribute to ECM formation by upregulating the expression of fibrosis-related genes in a TGF-β pathway-dependent manner. iCAFs promote the NF-κB pathway by IL1R-mediated IL1 signaling followed by LIF induction, leading to autocrine activation of JAK–STAT signaling to maintain the phenotype. Furthermore, Tuveson and colleagues revealed the presence of antigen-presenting CAFs (apCAFs) expressing MHC class II genes, distinct from myCAFs and iCAFs, by scRNA-seq and immunohistochemical staining of PDAC tissue [43]. In antigen-presenting cells such as dendritic cells, MHC class II molecules are usually also expressed together with co-stimulatory molecules required for induction of clonal proliferation of CD4-positive T cells, but apCAFs did not express co-stimulatory molecules [43,44]. Thus, the function of apCAFs in tumor immunity remains unclear.

Using a genetically engineered mouse model (GEMM) of PDAC, Hosein et al. performed scRNA-seq and revealed that the heterogeneity of tumors shifts with PDAC progression [45]. The results identified three CAF subtypes (FB1, FB2, and FB3) within normal and early PDAC but only two (FB1 and FB3) in advanced late-stage PDAC. FB1 was characterized by the expression of markers such as IL6, CCL2, CL7, CXCL12, and PDGFRA, while FB3 was characterized by the expression of myofibroblast markers ACTA2 and TAGLN. A portion of FB3 also expressed multiple MHC-II-related genes, suggesting that FB3 functions in antigen processing and presentation through the MHC-II pathway and complement activation. FB1 and FB3 were reported by Tuveson et al. to correspond to iCAFs and myCAFs, respectively, and the identification of CAFs expressing MHC-II-related genes supports the existence of apCAFs. Dominguez et al. also performed scRNA-seq using a GEMM of PDAC to classify CAF subtypes and used pseudo-time analysis to investigate how CAF populations change as the tumor progresses [39]. They reported that two distinct clusters of fibroblasts exist in the normal pancreas and that with tumor progression, each follows a distinct differentiation route to form two distinct CAF clusters. Moreover, pathway analysis revealed that one of the two clusters is mediated by IL1 and TNFα signaling, while another cluster is strongly enriched in the TGF-β fibroblast gene signature, which may correspond to the previously reported iCAFs and myCAFs. Thus, CAF subpopulations, such as myCAFs and iCAFs, have been identified in several single-cell analysis cohorts of human and mouse PDAC, and a consensus has been reached that there are two major subtypes of CAFs in PDAC tissue. Furthermore, scRNA-seq of breast and lung cancers has also identified CAF subtypes corresponding to myCAFs and iCAFs [46,47], and it is generally accepted that myCAFs are associated with ECM signatures, whereas iCAFs are characterized by secretory and inflammatory signatures in various cancer types [48]. In addition, there is common evidence of apCAFs expressing genes involved in antigen presentation, such as *Cd74*, *H2-Ab1*, and *Saa3*, and some reports have shown that the apCAF subtype shares an overlapping transcriptional signature with mesothelial cells [39,49,50].

## 3. Tumor-Promoting and Immunosuppressive CAF Subtypes

The potential role of CAFs in PDAC is to accumulate dense ECM and create a physical barrier that causes high interstitial pressure [51,52]. It serves as a scaffold to support cancer cell proliferation, migration, and invasion. Furthermore, the ECM induces the disruption of tumor vasculature, preventing the penetration of therapeutic drugs and antibodies [53]. Therefore, various stroma-targeting therapies have been developed, including SMO inhibitors that block the SHH signaling pathway and deplete the ECM by targeting αSMA-positive CAFs [23,54]. However, these stroma-targeting therapies have failed in clinical trials, resulting in a rather poor prognosis [18]. As described above, with the advent of single-cell analysis, the full picture of the heterogeneity of CAFs in PDAC tissues is gradually becoming clear. However, it remains uncertain how these three CAF subtypes function in cancer progression, as well as their direct function in each subtype and their indirect function via tumor immunity.

Considering that targeting αSMA-positive CAFs increased the tumor aggressiveness and worsened the prognosis, it is conceivable that myCAFs, characterized by high αSMA expression and ECM production, may have tumor suppressive functions. IL1 and TGF-β secreted by PDAC cells antagonize each other and define CAF subtypes such as iCAFs and myCAFs, which have the plasticity to change the phenotype depending on microenvironmental factors. Interestingly, inhibition of the JAK–STAT pathway, an intrinsic pathway of iCAFs, in the PDAC-transplanted mouse model shifted iCAFs to myCAFs and suppressed cancer cell proliferation, while ECM deposition increased. This suggests that iCAFs have tumor-promoting functions. However, the result was obtained by xenograft models using immunodeficient mice. Since JAK–STAT inhibitors may also target the proliferation and activity of cytotoxic T cells, the results of tumor immunity are unclear. CAFs have been shown to secrete immunosuppressive ligands such as TGF-β [55,56,57,58] and CXCL12 [13,59,60], which can prevent cytotoxic T-cell activity and migration into the tumor and recruit immunosuppressive populations such as MDSCs and neutrophils [61,62,63]. Evidence has shown that CAFs play an important role in the formation of immunosuppressive TMEs, but the influence of each CAF subtype on tumor immunity is controversial. iCAFs have been shown to be the main producers of immunosuppressive ligands such as CXCL12, IL6, CXCL1, and granulocyte colony-stimulating factor (G-CSF) [43,64,65,66]. IL6 derived from CAFs inhibits natural killer cell activity and activates STAT3 in pancreatic cancer cells [67]. In addition, IL6 derived from CAFs has been reported to promote immunosuppression by accelerating the differentiation of monocyte precursors into MDSCs [68,69]. Combination therapy with IL6 and PD-L1 antibody administered based on these results suppressed tumor progression and prolonged overall survival in a GEMM of pancreatic cancer (*Pdx1-Cre*, *lox-stop-lox-KrasG12D*/+, *lox-stop-lox-Trp53R270H/+*, and *Brca2^lox/lox^*) [70].

CXCL12, also known as stromal cell-derived factor 1 (SDF1), is a major ligand for CXCR4, and the CXCL12–CXCR4 axis promotes pancreatic cancer development, invasion, and metastasis [71,72]. In addition, inhibition of the interaction between CXCL12 and CXCR4 promoted T cell accumulation in central tumor areas and increased the effect of immune checkpoint blockade, suggesting that CXCL12 promotes spatial elimination of T cells [13]. Therefore, a phase I study was conducted to evaluate the safety of plerixafor (AMD3100), a specific CXCR4 inhibitor, in patients with advanced pancreatic, ovarian, and colorectal cancer (NCT 02179970). The results showed that a continuous administration of plerixafor induced the accumulation of intratumoral CD8+ cells and natural killer cells in pancreatic and colorectal cancer patients [73]. However, a phase II study with the CXCR4 antagonist BL-8040 (motixafortide) showed that BL-8040 increased the tumor infiltration of CD8+ T cells and decreased MDSCs and Tregs (NCT02826486) [74]. These observations suggest that iCAFs play a central role in the immunosuppression of PDAC TME.

myCAFs secrete TGF-β and enhance ECM formation, while the depletion of α-SMA+ cells leads to an increase in Tregs [19]. Therefore, myCAFs may express high levels of α-SMA promote antitumor immunity through direct or indirect mechanisms. However, several studies using scRNA-seq have reported that myCAFs are involved in tumor immunosuppression. Leucine-rich repeat containing 15 (LRRC15) has been reported to be expressed in the CAFs of many solid tumors, including pancreatic cancer, and in a subset of mesenchymal cancer cells [75], and scRNA-seq of PDAC mouse models revealed that LRRC15 is expressed in TGF-β-driven CAFs that may correspond to myCAFs [39]. Furthermore, clinical trials of immunotherapy in several cancer types, including bladder cancer, and non-small cell lung cancer, have shown that elevated levels of a LRRC15 + CAF signature correlated with poor response to anti-PD-L1 therapy. However, it is unclear how LRRC15+ CAFs are involved in the efficacy of anti-PD-L1 therapy. Single-cell analysis of human breast cancer revealed that the abundance of myCAF subgroups, namely ecm-myCAFs and TGF-β-myCAFs, was significantly correlated with the immunosuppressive environment, while the abundance of iCAF subgroups, namely detox-iCAFs and IL-iCAFs, was not. In addition, ecm-myCAF and TGF-β-myCAF subgroups were enriched in tumors with a high proportion of PD-1+CTLA4+TIGIT+ CD4+ T lymphocytes and a low proportion of CD8+ T lymphocytes. These results contradict the findings that depletion of α-SMA+ cells increased regulatory T cells. However, the expression levels of α-SMA are heterogeneous among CAFs, and these ecm-myCAF and TGF-β-myCAF subgroups were subclusters within the FAP+ fibroblast population and are not included in the FAP-fibroblast population.

apCAFs express MHC II molecules but lack the classical costimulatory molecules (e.g., CD40, CD80, and CD86) required to induce activation and clonal expansion of CD4+ T cells after T cell receptor (TCR) ligation [43]; therefore, their role in tumor immunity has not been verified. apCAFs have been shown to have a genetic signature similar to that of mesothelial cells identified in normal pancreas by single-cell analysis [39,49,50]. Subsequent further analyses integrating multiple scRNA-seq studies and a lineage tracking assay revealed that apCAFs originate from mesothelial cells [76]. Furthermore, it was found that in normal pancreas, mesothelial cells form a thin membrane covering the margin of the pancreas, but as PDAC progresses, TGF-β and IL1 derived from PDAC cells reduce the expression of mesothelial genes (*Msln*, *Upk3b*, *Ezr*, and *Nkain4*) in normal mesothelial cells and change their phenotype to apCAF. However, the lack of co-stimulatory molecules on antigen presenting cells has been reported to lead to T cell anergy and induction of regulatory T cells [77,78,79]. Consistent with these reports, apCAFs activated CD4+ T cells and promoted their differentiation into FOXP3+ Tregs, directly contributing to tumor immunosuppression.

## 4. Tumor-Suppressive and Tumor Differentiation-Related CAF Subtypes

CAF subtypes that contribute to cancer tumor progression have been extensively studied, but few reports have focused on CAFs that restrain tumor progression. In a PDAC mouse model, selective genetic depletion of αSMA+ CAFs or suppression of CAFs by a pharmacological blockade of SHH signaling increased the proportion of poorly differentiated forms and promoted cancer progression by causing an escape from immune surveillance with increased Treg infiltration and vascular alterations [19,20]. These reports suggest that there are some tumor suppressive populations among CAFs in PDAC. PDAC patients with higher αSMA scores or higher tumor stromal density had better overall survival [19,80]. The findings that inhibition of SHH signaling reduces ECM deposition and αSMA-positive cells [8] suggests that tumor suppressive CAFs are involved among αSMA-positive CAFs. However, αSMA is expressed in the majority of CAFs, although its expression intensity varies. Furthermore, it is expressed by pericytes, smooth muscle cells comprising the vessel wall and smooth muscle layer, and myoepithelial cells in almost all tissues, limiting the use of αSMA in classifying tumor-suppressive CAFs.

Meflin is a glycosylphosphatidylinositol-anchored protein and is considered as a potential marker for mesenchymal stromal cells [81]. Mizutani et al. reported that meflin-positive CAFs are among tumor-suppressive CAFs [82]. In situ hybridization findings of human PDAC tissue showed that the involvement of meflin-positive CAFs correlated with favorable patient outcomes. The knockout of *Islr* (immunoglobulin superfamily containing a leucine-rich repeat) encoding meflin in PDAC mouse models resulted in tumor progression and poorly differentiated tumors. Furthermore, meflin-positive CAFs are also positive for GLI1, a transcription factor important for SHH signaling, and weakly express αSMA, suggesting that the tumor promotion may be caused by the simultaneous depletion of meflin-positive CAFs by depletion of αSMA-positive CAFs and pharmacological blockade of SHH signaling.

**Figure 2:**
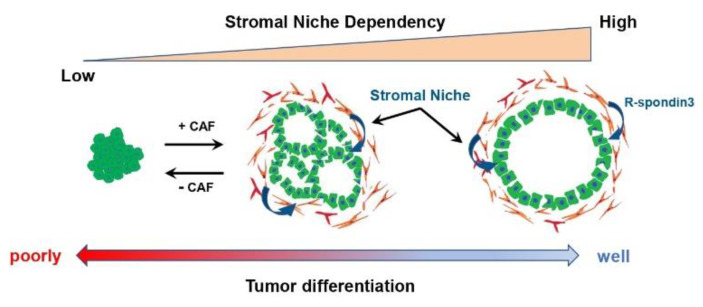
The relationship between the tumor differentiation grade and the dependency on stromal factors derived from CAFs in PDAC.

In a GEMM of PDAC, an increased proportion of poorly differentiated PDAC cells upon CAF suppression was reported, suggesting that CAFs are involved in regulating the PDAC phenotype, such as differentiation grade. In bladder and colorectal cancer, stromal cells were reported to maintain cancer cell differentiation through SHH-mediated BMP secretion [83,84]. In PDAC, differentiation grade remains one of the gold standards for the clinical characterization of tumors, with well-differentiated tumors having a good prognosis and poorly differentiated tumors have a poor prognosis associated with high invasive and metastatic potential [85]. In addition, several recently reported RNA expression analyses using bulk tumor tissue revealed the existence of two major molecular subtypes with distinct patient prognoses and biological characteristics [86]. While the “classic” or “progenitor” subtype defined by expressing some epithelial markers such as CDH1 and GATA6 and good prognosis, the “basal-like”, “squamous”, or “quasi-mesenchymal” subtypes exhibit mesenchymal marker expression and more aggressive behavior, with a poor prognosis [86,87,88]. However, the relationship between these PDAC subtypes and CAFs has not been clarified. One reason for this is that preclinical models reflecting these subtypes are limited, because all pancreatic cancer cell lines conventionally used in two-dimensional culture are classified as “basal-like” subtypes [86]. However, with the advent of the PDAC organoid model [89], various PDAC subtypes can now be established and cultured, and the relationship between PDAC subtypes and CAFs can be evaluated. Raghavan et al. identified a PDAC subtype that showed intermediate gene expression signatures between “basal-like” and “classical” and co-expressed features of both programs to varying degrees using the PDAC organoid model [90]. Furthermore, they revealed that the intermediate subtype of PDAC alters the expression signature through tumor microenvironmental factors. Conversely, we used a coculture model of PDAC organoids and CAFs to assess the relationship between the differentiation grades of PDAC and CAFs [91]. The results revealed that well-differentiated PDAC organoids categorized as the classical type are strongly dependent on CAFs for their proliferation and for the maintenance of the differentiated morphology, the ductal structure. Furthermore, moderately differentiated PDAC organoids exhibited differentiation plasticity that changes the differentiation depending on the presence or absence of CAFs, suggesting that CAFs maintain the differentiation state of PDAC by supplying microenvironmental factors such as R-spondin 3 to the PDAC cells (Figure 2). Consistent with the results ablating the stroma in the PDAC mouse model, these findings suggest that inhibition of CAFs may risk switching the PDAC phenotype from the differentiated to poorly differentiated and leading to a worse prognosis [19,20]. Moreover, poorly differentiated PDAC organoids and pancreatic cancer cell lines did not show plasticity to switch to a well differentiated state even in the presence of CAFs. Therefore, this finding indicates that the PDAC phenotype that switches according to the suppression of CAFs cannot be assessed in transplanted mouse models using pancreatic cancer cell lines that do not have differentiation plasticity. As mentioned above, there is heterogeneity within CAFs, mainly iCAFs, myCAFs, and apCAFs, but it is unclear which subtype of CAFs is responsible for the maintenance of the PDAC differentiation state. In immunohistochemical staining, CAFs in close proximity to PDAC cells expressed R-spondin 3, along with αSMA. However, in normal intestinal epithelium, myofibroblasts were reported to maintain the homeostasis of gastrointestinal epithelial stem cells via the secretion of R-spondin 3 [92,93]. Given these reports and the fact that αSMA-positive CAFs were the target of studies in which CAF suppression increased the proportion of poorly differentiated PDACs in mouse models, it is suggested that the CAF population involved in maintaining the differentiated state may belong to myCAFs.

Many tumor-promoting functions of CAFs have been reported, including the formation of physical barriers to drugs via the ECM, the promotion of cancer cell invasion and metastatic potential through cancer–stroma interactions, and the recruitment of immunosuppressor cells. Therefore, many clinical trials have attempted to improve patient survival by targeting CAF-related factors. However, as shown above, recent data have demonstrated a tumor-suppressing role for CAFs [19,20,54], and accordingly, an anti-CAF therapy for PDAC is being developed to revert active fibroblasts back to quiescent fibroblasts rather than eliminate CAFs altogether. All-trans retinoic acid (ATRA) activates retinoic acid receptors and is a major activator of retinoic acid signaling [94], and treatment with ATRA converted CAFs from an activated state to a quiescent state, reducing the stroma and inhibiting cancer cell proliferation in KPC mice. Furthermore, ATRA administration increased CD8+ T cells in juxtatumoral compartments in KPC mice [59]. On the basis of these results, a phase 2 study (ATRA/gemcitabine/nab-paclitaxel) is currently underway (NCT04241276). Activation of a vitamin D receptor in CAFs suppresses inflammation and fibrosis, and calcipotriol (vitamin D3 analog) was reported to be effective as a treatment for PDAC [95]. However, recent studies have reported that calcipotriol has immunosuppressive effects on CD8+ T cells [96]. Thus, several therapies have been developed to return CAFs from an activated state to a quiescent state, but these do not consider the heterogeneity of CAFs. It is unclear how the transition of CAFs to a quiescent state by these therapies affects the phenotype of PDAC cells.

Accordingly, it is preferable to develop therapies that target only tumor-promoting CAFs, considering the presence of tumor-suppressing CAF subtypes and CAF subtypes that retain PDAC differentiation. However, specific markers to identify CAF subtypes such as iCAFs, myCAFs, and apCAFs are still lacking. The depletion of Fap-positive cells via transgenic diphtheria toxin receptor (DTR) expression demonstrated reduced muscle mass and hematopoietic cells, while tumor growth was reduced in PDAC mouse models [97]. Some of these targeted CAF markers are also expressed in other normal cell types, indicating that therapeutic targeting to specific CAF subtypes is difficult as a treatment approach. Consequently, targeted therapies that directly block immunosuppressive ligands secreted by CAFs are also being developed. The CXCL12–CXCR4 axis has been shown to promote disease progression and immunosuppression in breast cancer [46,60]. In PDAC, inhibition of CXCR4 by AMD3100 increased T-cell infiltration and improved the efficacy of checkpoint inhibitors [13]. Further, AMD3100 treatment attenuates pancreatic cancer development in KPC mice. Along these lines, the inhibition of CXCR4 signaling in CAFs has been shown to reduce immunosuppressive cell populations and increase the efficacy of immunotherapy [73].

Another strategy is to design therapies that exploit CAF plasticity by shifting the tumor-promoting CAF subtype to a tumor-suppressing subtype. Recent studies have shown that CAF subtypes are dynamic and alter each other in response to microenvironmental factors such as culture conditions and surrounding cells [37,43]. Therefore, it is ideal to develop therapies that switch from tumor-promoting to tumor-suppressive subtypes. Of the three CAF subtypes, myCAFs, iCAFs, and apCAFs, iCAFs, in particular, have been shown to have tumor-promoting activity via IL1–JAK–STAT signaling [37,38,43]. Accordingly, targeting iCAFs with the JAK inhibitor AZD1480 shifted iCAFs to myCAFs and increased ECM deposition in a PDAC mouse model [38]. However, while JAK inhibitors are effective at targeting cancer cells and iCAFs, they also have the potential to target the proliferation and activity of cytotoxic T cells, making their combination with immunotherapy problematic [48]. To address this, a clinical trial is now underway to add an IL1 receptor antagonist (anakinra) to chemotherapy for PDAC, which may limit the tumor-promoting activity of iCAFs (NCT04926467). However, homeodomain transcription factor paired-related homeobox 1 (PRRX1) regulates epithelial-to-mesenchymal transition (EMT) [98]. PRRX1 is considered a driver of cellular plasticity during acinar-to-ductal metaplasia (ADM) and carcinogenesis [99]. Furthermore, PRRX1 has been found to be expressed in CAFs in mouse and human PDAC tissues. CAFs deficient in PRRX1 have reduced cell plasticity and differentiate into myCAFs [100]. Therefore, targeting PRRX1 may be a potential strategy to reprogram CAFs from a tumor-promoting subtype to a tumor-suppressing subtype.

## 5. Conclusions

scRNA-seq has revealed the heterogeneity of CAFs with different functional subpopulations in PDAC. The heterogeneity of immune cells in PDAC TME is also emerging, and the relationship between CAF subtypes and the immune microenvironment is becoming clearer. However, some of the most used CAF markers are also expressed on other cell types, and there is a lack of known specific surface markers that can sort out the subpopulations identified by scRNA-seq; therefore, it remains unclear what functions these subpopulations actually have. Furthermore, tumor-suppressive CAFs and CAFs that maintain PDAC differentiation have not been identified as clusters by scRNA-seq. In the future, we hope to overcome these challenges and specifically evaluate the actual function of each subpopulation to develop novel targeted therapies that alter the tumor immune microenvironment by targeting specific CAF subtypes in PDAC.

## Data Availability

The data can be shared up on request.
